# Glycerol, a possible new player in the biology of trypanosomes

**DOI:** 10.1371/journal.ppat.1010035

**Published:** 2021-12-02

**Authors:** Frédéric Bringaud, Nicolas Plazolles, Erika Pineda, Corinne Asencio, Oriana Villafraz, Yoann Millerioux, Loïc Rivière, Emmanuel Tetaud

**Affiliations:** Microbiologie Fondamentale et Pathogénicité (MFP), UMR 5234, Bordeaux University, CNRS, Bordeaux, France; University at Buffalo School of Medicine and Biomedical Sciences, UNITED STATES

## Introduction

*Trypanosoma brucei* is a unicellular eukaryote that causes human African trypanosomiasis, also known as sleeping sickness [1]. Parasite transmission between mammals is ensured by a haematophagous insect vector of the genus *Glossina*, also called the tsetse fly. Trypanosomes adapt to their natural hosts, in particular to the available carbon sources required to fuel central metabolism and to produce ATP. For instance, in the digestive tract of the insect vector, procyclic forms (PCFs) of *T*. *brucei* use proline abundantly present in the insect [2]. It has recently been proposed that other carbon sources may also be required for the parasite in the fly, such as glucose at the onset of infection or intermediates of the tricarboxylic acid cycle [3,4]. By contrast, the bloodstream forms (BSF) rely on glucose present at homeostatic levels in all mammalian fluids [2]. In addition to proline and glucose, glycerol can also fuel central carbon metabolism of the parasite, but its relevance has received little attention until recently. This Pearl article will highlight recent data on glycerol metabolism and their implications for understanding trypanosome biology.

### Procyclic trypanosomes (PCF) prefer glycerol over glucose

As mentioned above, PCF evolve in the glucose-depleted midgut of their insect vector, where they rely on proline [5,6], but, nevertheless, use glucose as the primary carbon source and down-regulate proline consumption up to 7 times under *in vitro* conditions [7,8]. The molecular mechanism of this glucose preference over proline is currently unknown. Surprisingly, the parasite also developed an absolute preference for glycerol over glucose, already described in the 1960s by Ryley [9] and recently revisited by our group [10]. Indeed, glucose is not consumed as long as glycerol is present in the medium. As far as we know, *T*. *brucei* is the only unicellular eukaryote to date reported to prefer a nonglycolytic carbon source to glucose.

**Fig 1:**
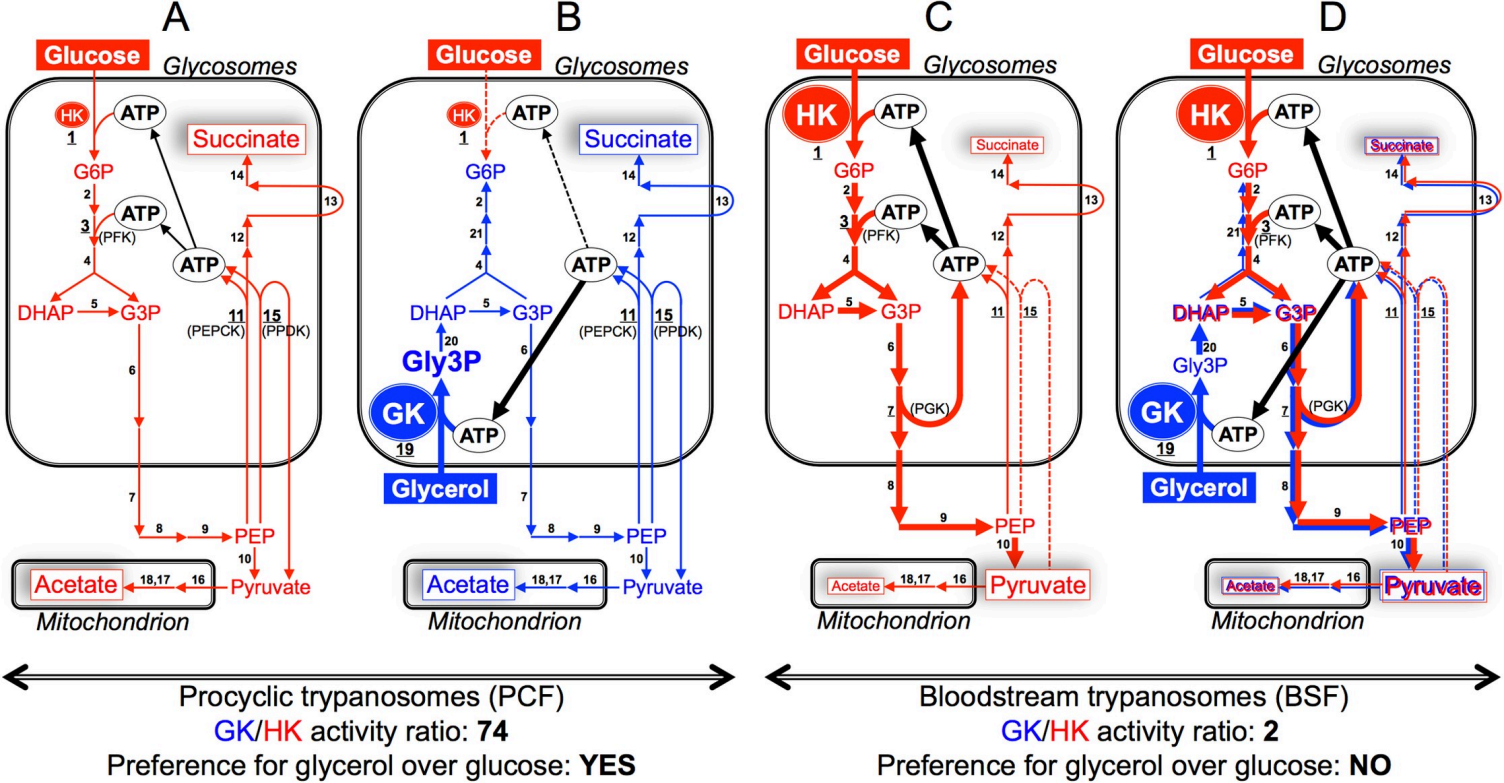
Mechanism of metabolic contest between glycosomal HK and GK. Pathways leading to excretion of end products (succinate, acetate, and pyruvate, highlighted in white rectangle) from metabolism of glucose and glycerol are indicated in red and blue, respectively. The production and consumption of ATP within the glycosomes are indicated and maintenance of the glycosomal ATP balance is highlighted by black arrows. The thickness of the arrows reflects metabolic fluxes and metabolic steps detected only at relatively very low activity or not at all are represented by dotted lines. The GK/HK activity ratio for procyclic and bloodstream forms is indicated below the corresponding schemes (A–B and C–D, respectively). Enzymes are (those underlined are glycosomal kinases producing or consuming ATP) the following: 1, hexokinase (HK); 2, glucose-6-phosphate isomerase; 3, phosphofructokinase; 4, aldolase; 5, triose-phosphate isomerase; 6, glyceraldehyde-3-phosphate dehydrogenase; 7, phosphoglycerate kinase (PGK); 8, phosphoglycerate mutase; 9, enolase; 10, pyruvate kinase; 11, phosphoenolpyruvate carboxykinase (PEPCK); 12, malate dehydrogenase; 13, fumarase; 14, NADH-dependent fumarate reductase; 15, pyruvate phosphate dikinase (PPDK); 16, pyruvate dehydrogenase complex; 17, acetate:succinate CoA-transferase; 18, acetyl-CoA thioesterase; 19, glycerol kinase (GK); 20, glycerol-3-phosphate dehydrogenase; 21, fructose-1,6-bisphosphatase. Abbreviations are: BSF, bloodstream forms; DHAP, dihydroxyacetone phosphate; G3P, glyceraldehyde 3-phosphate; G6P, glucose 6-phosphate; Gly3P, glycerol 3-phosphate; PCF, procyclic form; PEP, phosphoenolpyruvate.

We characterised the molecular mechanism of this glycerol preference, which was called “metabolic contest” since it is based on competition between 2 kinases (hexokinase [HK] and glycerol kinase [GK]) for the same substrate (ATP) [10]. When glucose and glycerol enter the cells via glucose transporters and aquaglyceroporins, they are first phosphorylated to glucose 6-phosphate (G6P) and glycerol 3-phosphate (Gly3P) by HK (step 1 in Fig 1) and GK (step 19), respectively. The phospho group prevents G6P, Gly3P, and the derived phosphorylated metabolites from leaving the cell through transport processes, participates in ATP production, and facilitates enzyme binding and activity. In trypanosomes, HK and GK, as well as most other enzymes involved in glycolysis and glycerol metabolism, are located in peroxisome-related organelles, named glycosomes, which show limited or no nucleotide exchange with the cytosol on a metabolic timescale. Therefore, consumption and production of ATP are tightly balanced within the organelle [11], with each ATP molecule required to supply GK and HK being regenerated by phosphoenolpyruvate carboxykinase (PEPCK, step 11) and pyruvate phosphate dikinase (PPDK, step 15) in PCF glycosomes (Fig 1A and 1B). Hence, the limitation of the glycosomal ATP pool available to glycosomal kinases offers a situation where a significant excess of one kinase (here GK) can theoretically abolish the metabolic flux through another one (here HK). In this context, the very large excess of GK activity compared to HK activity (74-fold excess) explains the absolute glycerol preference confirmed by genetic manipulations and metabolic approaches [10]. The competition of given enzymes for a common substrate is a well-known process used to finely tune metabolic fluxes, in particular at metabolic branch points [12]. However, this is the first example of a complete repression of one enzymatic catalysis (HK) by the large excess of another one (GK), as a mechanism to control nutrient utilisation.

Metabolic contest is a new concept to describe metabolic choices that resembles the well-characterised catabolic repression or carbon catabolite repression observed in prokaryotes, yeasts, and fungi, albeit based on a completely different molecular mechanism. The mechanisms by which carbon catabolite repression is imposed are quite variable. They follow a general rule, however, with complex sensory systems relying mostly on protein kinases and phosphatases [13], which include the sugar uptake phosphotransferase system characterised in the 1960s [14]. The advantage of the metabolic contest *versus* carbon catabolite repression mechanism is mainly an immediate switch to the less preferred carbon source when the preferred one is exhausted. Theoretically, a metabolic contest could be found in any organism provided that 3 conditions are met, *i.e.*, (i) the sequestration of both metabolic pathways in the same subcellular compartment; (ii) the competition for the same substrate between 2 enzymes, one in each pathway; and (iii) an unbalanced activity between the competing enzymes.

The physiological role of glycerol metabolism *in vivo* in the fly is still an open question. The preference of glycerol over glucose is based on the huge overexpression of GK, which could be reduced by at least 90% without affecting the glycerol metabolic flux [10]. Incidentally, glycerol also induces a 2.3-fold reduction of GK expression, without affecting metabolic contest, suggesting that the very high GK level in glucose-rich conditions is not compatible with glycerol-rich conditions, may be due to accumulation of Gly3P or other downstream metabolites, and/or a sharp decrease in the glycosomal ATP/ADP ratio [10]. It is also noteworthy that, in *T*. *brucei*, GK is encoded by 5 duplicated *GK* genes, while the genome of almost all other trypanosomatids contains a single *GK* gene, suggesting that *GK* gene duplication was positively selected in *T*. *brucei* to allow the preference for glycerol. Although, one cannot exclude that the high GK level has other reasons (see next section).

Glycerol has also been shown in vitro to prevent differentiation of early PCF, which expressed the surface protein GPEET, to late PCF, which are GPEET negative [15]. GPEET is expressed during midgut infection, and its down-regulation correlates with trypanosomes crossing the peritrophic matrix and colonising the ectoperitrophic space [16], suggesting that glycerol may be present in the digestive tract of the insect to prevent down-regulation of GPEET expression. Unfortunately, with the exception of amino acids [5], the metabolite content in the midgut and other organs of the tsetse has not been studied so far. Thus, further investigation should be done to determine whether glycerol plays a role in the biology of trypanosomes in the insect vector.

### Bloodstream trypanosomes (BSF) also metabolise glycerol efficiently

The topology of the central carbon metabolic network is similar between PCF and BSF, with one main exception, *i.e.*, the cytosolic and glycosomal localisation of phosphoglycerate kinase (PGK, step 7), respectively [17]. Another major difference resides in the 10-fold higher glycolytic flux in BSF (for example, HK activity is up-regulated 28-fold [10]), with pyruvate representing up to 95% of the end products excreted from metabolism of glucose [18] (Fig 1C), while PCF mainly convert glucose to excreted acetate and succinate [19] (Fig 1A). Consequently, PGK is responsible for the regeneration of glycosomal ATP in BSF (Fig 1C), rather than PEPCK/PPDK as mentioned above for PCF (Fig 1A). It is noteworthy that the high glycolytic flux described in *T*. *brucei* BSF, as well as in plant-infecting *Phytomonas* spp., remains the exception in the trypanosomatid world as most of the trypanosomatid parasitic forms deve-loped a “procyclic-like” form of glycolysis, *i.e.*, relative low glycolytic flux and pyruvate being further metabolised in the glycosomes and the mitochondrion [2,20].

Because of the intronless and polycistronic expression nature of trypanosome genes, differential expression of glycolytic enzymes should be controlled posttranscriptionally [21]. No RNA-binding protein directly involved in this process has been identified so far; however, knockout of a major type I protein arginine methyltransferase (PMRT1) induced changes that resemble the metabolic remodelling that occurs during *T*. *brucei* life cycle progression to PCF, including down-regulation of HK expression [22].

Besides glucose, glycerol utilisation as substrate for ATP production was first proposed decades ago [23], but the possibility of prolonged growth with this substrate has not been addressed until recently. In fact, glycerol was considered a poison, because of its high toxicity for BSF grown in anaerobiosis or in combination with drugs targeting the alternative oxidase [24], since glycerol prevents ATP production by reversal of the GK that must occur under these conditions to maintain the glycosomal ATP/ADP balance. However, in 2018, 2 groups independently reported that glycerol can support growth of BSF *in vitro*, so far believed to be exclusively dependent on glucose for growth [25,26]. This resonates with the recent discovery that the parasite colonises and propagates in the skin and adipose tissues of its mammalian hosts, where adipocytes produce significant amounts of glycerol [27–29], suggesting that glycerol metabolism may play a role for the *in vivo* development of BSF. It is to be noted that the low GK/HK activity ratio (2.2) provides a rational explanation for the absence of metabolic contest for ATP between GK and HK, resulting in concomitant consumption of glucose and glycerol when both are present (Fig 1D). Indeed, although GK is constitutively expressed in *T*. *brucei* grown under glucose-rich conditions, HK activity is 28-fold higher in BSF than in PCF, as mentioned above [10].

The glycerol-dependent reduction of GK expression mentioned above for PCF also occurs in BSF [25]. Considering the absence of glycerol preference in this parasitic form, this observation suggests that the reason for the large excess of GK level may be related to glycolysis. Indeed, high GK activity may be required for production of glycerol from glycolysis, for instance, when oxygen is limiting, since GK’s specific activity for glycerol production is much lower than that for glycerol phosphorylation [30]. However, it cannot be excluded that extravascular trypanosomes require high GK activity to adapt to glycerol-rich tissue.

The relatively low abundance of glycerol in the bloodstream (50 to 100 μM) [31,32] compared to glucose (5 mM) and the slight preference of BSF for glucose over glycerol [25], imply that glucose is indeed the main source of ATP for BSF in mammalian fluids. However, in the interstitial fluid of mammalian tissues, the extravascular trypanosomes meet a glucose-rich environment (in the range of 3 mM) containing from 0.2 to 3 mM glycerol, depending on the report and the tissues analysed [31,32], suggesting that trypanosomes may adapt and benefit from this potentially glycerol-rich environment. In this context, the resulting *in vivo* glycerol gradient between the intra- and extravascular compartments could influence the parasite tropism to particular tissues *via* specific sensing pathways, such as the social motility phenomenon described in PCF in the insect midgut [33]. The ongoing analyses of these recently discovered extravascular trypanosomes will certainly reveal fascinating new features, especially on carbon source utilisation. Although the exact role of glycerol metabolism in BSF *in vivo* is not understood yet, these data open novel avenues for developing new diagnostic tools and/or treatments based on unexplored molecular targets.
